# A diagnostic circulating miRNA signature as orchestrator of cell invasion via TKS4/TKS5/EFHD2 modulation in human gliomas

**DOI:** 10.1186/s13046-023-02639-8

**Published:** 2023-03-17

**Authors:** Ana Belén Díaz Méndez, Andrea Sacconi, Elisa Tremante, Valentina Lulli, Valentina Caprara, Laura Rosanò, Frauke Goeman, Mariantonia Carosi, Marta Di Giuliani, Giulia Vari, Antonio Silvani, Bianca Pollo, Carlo Garufi, Sara Ramponi, Giorgia Simonetti, Emilio Ciusani, Chiara Mandoj, Stefano Scalera, Veronica Villani, Agnese Po, Elisabetta Ferretti, Giulia Regazzo, Maria Giulia Rizzo

**Affiliations:** 1 Department of Research, Advanced Diagnostics and Technological Innovation, Translational Oncology Research Unit, IRCCS Regina Elena National Cancer Institute, Via Elio Chianesi 53, 00144 Rome, Italy; 2 Biostatistics and Bioinformatics Unit, Clinical Trial Center, IRCCS Regina Elena National Cancer Institute, Rome, Italy; 3 Department of Hematology, Oncology and Molecular Medicine, Istituto Superiore di Sanità, Rome, Italy; 4 Preclinical Models and New Therapeutic Agents Unit, IRCCS Regina Elena National Cancer Institute, Rome, Italy; 5 Institute of Molecular Biology and Pathology (IBPM), National Research Council (CNR), Rome, Italy; 6 SAFU Unit, IRCCS Regina Elena National Cancer Institute, Rome, Italy; 7 Pathology Unit, IRCCS Regina Elena National Cancer Institute, Rome, Italy; 8 PhD Program in Molecular Medicine, Department of Molecular Medicine, Sapienza University of Rome, Rome, Italy; 9 Neuro-Oncology Unit, Fondazione IRCCS Istituto Neurologico Carlo Besta, Milan, Italy; 10 Medical-Oncology Unit, San Camillo Forlanini Hospital, Rome, Italy; 11 Clinical Pathology Unit, IRCCS Regina Elena National Cancer Institute, Rome, Italy; 12 PhD Program in Cellular and Molecular Biology, Department of Biology, University of Rome “Tor Vergata”, Rome, Italy; 13 Neuro-Oncology Unit, IRCCS Regina Elena National Cancer Institute, Rome, Italy; 14 Department of Experimental Medicine, Sapienza University, Rome, Italy

**Keywords:** Glioma, IDH, Circulating microRNA, Diagnosis, Invasion, TKS4, TKS5, EFHD2

## Abstract

**Background:**

Altered microRNA profiles have been observed not only in tumour tissues but also in biofluids, where they circulate in a stable form thus representing interesting biomarker candidates. This study aimed to identify a microRNA signature as a non-invasive biomarker and to investigate its impact on glioma biology.

**Methods:**

MicroRNAs were selected using a global expression profile in preoperative serum samples from 37 glioma patients. Comparison between serum samples from age and gender-matched controls was performed by using the droplet digital PCR. The ROC curve and Kaplan-Meier survival analyses were used to evaluate the diagnostic/prognostic values. The functional role of the identified signature was assessed by gain/loss of function strategies in glioma cells.

**Results:**

A three-microRNA signature (miR-1-3p/−26a-1-3p/−487b-3p) was differentially expressed in the serum of patients according to the isocitrate dehydrogenase (IDH) genes mutation status and correlated with both patient Overall and Progression Free Survival. The identified signature was also downregulated in the serum of patients compared to controls. Consistent with these results, the signature expression and release in the conditioned medium of glioma cells was lower in IDH-wild type cells compared to the mutated counterpart. Furthermore, in silico analysis of glioma datasets showed a consistent deregulation of the signature according to the IDH mutation status in glioma tumour tissues. Ectopic expression of the signature negatively affects several glioma functions. Notably, it impacts the glioma invasive phenotype by directly targeting the invadopodia-related proteins TKS4, TKS5 and EFHD2.

**Conclusions:**

We identified a three microRNA signature as a promising complementary or even an independent non-invasive diagnostic/prognostic biomarker. The signature displays oncosuppressive functions in glioma cells and impacts on proteins crucial for migration and invasion, providing potential targets for therapeutic intervention.

**Supplementary Information:**

The online version contains supplementary material available at 10.1186/s13046-023-02639-8.

## Background

Gliomas, the most common intrinsic tumours of the brain, are deemed surgically challenging due to their anatomic location, diffusion and infiltrative nature, which are detrimental to brain functioning [1]. Gliomas can be classified into several molecular subtypes, many of which have a well-defined profile of driver mutations, such as those in the isocitrate dehydrogenase 1 (IDH1) or 2 (IDH2) genes [2]. This biomarker-driven classification is partly reflected in the 2021 update of the World Health Organization (WHO) classification of brain tumours and is further underlined in the cIMPACT-NOW guidelines [3]. This has resulted in the concept of an integrated histological and molecular diagnosis [4]. Among the key genetic events, the IDH mutations are noteworthy from both a diagnostic and a prognostic point of view [4]. Mutations in IDH genes, observed at high frequencies in diffuse astrocytomas, oligodendrogliomas and secondary glioblastomas, have a specific metabolic profile, a hypermethylated phenotype and a better prognosis [5].

Tissue biopsies are essential for establishing a diagnosis and a molecular profile. However, they are invasive procedures carrying a significant risk and may only capture a static snapshot of the disease that does not reflect the heterogeneous features of the tumour [6]. Conversely, liquid biopsies allow the assessment, with multiple sampling, of a wide landscape of molecular markers that the tumour sheds via body fluids, leading to a dynamic patient management and a personalized medicine approach [6].

MicroRNA (miRNAs), small non-coding RNAs, are important players in glioma biology, by facilitating or hampering tumour development [7]. MiRNA expression profiles are frequently altered not only in tumour tissues but also in biofluids of glioma patients and show great potential as non-invasive biomarkers due to their high stability in body fluids and a range of sensitivity and specificity of ~ 30–99% and ~ 70–100%, respectively [2]. Several studies described deregulated profiles of circulating miRNAs in the serum of glioma patients compared to healthy individuals and a correlation between some circulating-miRNAs and patient outcome has been observed [8, 9]. This suggests a potential diagnostic and prognostic value for circulating miRNAs. This could be particularly valuable in a brain tumour context where other nucleic acids, such as cell-free DNA (cfDNA), show lower or absent levels in patient blood compared to other solid tumours, probably due to a greater difficulty in crossing the blood brain barrier (BBB) compared to miRNAs, which have been shown to pass the barrier as extracellular vesicles load [2, 10, 11]. Yet, only a few studies take the opportunity to integrate the molecular profile of gliomas with circulating miRNA levels, where in many cases circulating miRNAs have been investigated as single biomarkers [2]. However, it is widely recognized that single miRNA profiles are less accurate as cancer biomarkers, mostly due to the multifactorial nature of the tumour and to the large number of targets for a single miRNA [9]. Thus, the evaluation of a multiple miRNA-signature could be a more valuable and reliable approach in identifying molecular markers that could mirror the complexity of the disease and that can play a meaningful role in glioma biology.

In this study, we identified, in accordance with the REporting MARKer (REMARK) guidelines [12], a three serum miRNA signature (miR-1, miR-26a-1 and miR-487b) as a sensitive and specific diagnostic and prognostic biomarker that impacts common targets crucial for glioma growth, survival and invasion. In particular, we have identified as novel direct targets of the miRNA signature, *SH3PXD2B*, *SH3PDX2A* and *EFHD2* genes, which encode for signalling-mediating proteins involved in cell adhesion and migration by controlling the invadopodium activity [13, 14], that may contribute to the IDH-wild type (IDH-wt) glioma invasiveness and provide potential targets for combined therapeutic interventions.

## Methods

### Patients and control subjects

From November 2014 to March 2019, a training cohort of 37 serum samples were collected from glioma patients with different IDH mutation status (27 patients with IDH wild-type and 10 patients with IDH mutation) at the Regina Elena National Cancer Institute (IRE). An additional set of 15 serum glioma samples were recruited from July 2020 to November 2021 at the Besta Institute and San Camillo Hospital. Healthy controls were recruited at the IRE from individuals seeking a routine health check-up, having no evidence of disease and with age, gender and ethnicity matched to the patients.

Information on patients and healthy subjects is shown in Table 1.

**Table 1:** Biologic and Clinic features of the patients and healthy subjects enrolled in the study

Characteristics	Small-RNA Seq cohort		Cohort for comparison gliomas vs controls	
	IDH-wt (*n =* 27)	IDH-mut (*n =* 10)	Gliomas (*n =* 15)	Healthy subjects (*n =* 15)
Age				
Median	59	47	50	51
Range	24–77	29–77	23–70	23–70
Gender				
Males	20	7	9	9
Females	7	3	6	6
Histology				
Glioblastoma, IDH-wt	25	–	9	–
Astrocytoma, IDH-mut	–	3	1	–
Oligodendroglioma	0	6	2	–
Glial neoplasia, unspecified	2	1	3	–
IDH mutation status				
wild type	27	0	8	–
IDH1 mutant	0	10	6	–
IDH2 mutant	0	0	1	–
1p19q status				
Non codeleted	27	3	13	–
Co-deleted	0	4	2	–
Unknown	0	3	0	–
MGMT promoter status				
Methylated	15	5	6	–
Unmethylated	12	3	7	–
Unknown	0	2	2	–

### Serum collection and processing

Blood samples of glioma patients were collected before surgery, radiotherapy or chemotherapy in BD Vacutainer serum tubes using a 21-gauge needle. The samples were kept at room temperature (RT) for 30–60 min and then centrifuged at RT for 20 min at 1200 g. The serum transferred into sterile cryovials was aliquoted and stored at − 80 °C until further analysis. RNA isolation, cDNA synthesis, qRT-PCR were performed as described [8].

### Droplet-digital PCR (ddPCR)

The cDNA was added to the reaction mix containing 2XddPCR Supermix for Probes (#1863010, Biorad) and TaqMan miRNA PCR primer probe set (Taqman miRNA Assay #4427975, ThermoFisher Scientific). The PCR mixes for each sample were loaded in a disposable DG8 cartridge (#1864008, Biorad) together with droplet generation Oil (#1863005, Biorad) and loaded in the QX200 droplet generator (#1864002, Biorad). Droplets were then transferred into a 96-well plate, the plate was heat-sealed with foil and then placed in a thermal cycler. An endpoint PCR was performed using the following conditions: 95 °C for 10 min, then 40 cycles at 95 °C for 15 sec and 60 °C for 1 min, and a final step at 98 °C for 10 min. After the PCR run, the 96-well plate was placed in the QX200 Droplet Reader (#1864003, Biorad) for detection. The percentage of positive droplets was calculated by the software QuantaSoft (Biorad). MiRNA expression was analysed calculating copies of miRNA/μL.

### cDNA library preparation

The small RNA-Seq library was prepared from RNA obtained from serum using the QIAseq miRNA Library Kit (#331502 Qiagen) following the manufacturer’s instructions. The quality of the resulting libraries was controlled with the Bioanalyzer and the High Sensitivity DNA Kit (#5067–4626, Agilent Technologies). The quantification of the libraries was performed with the Qubit fluorimeter and the dsDNA HS Assay Kit (#Q33230, ThermoFisher Scientific) and the pool of libraries by qPCR. The final pool was diluted to 4 nM, denatured and further diluted for sequencing following the manufacturer’s instructions.

### Small RNA sequencing analysis

A genome-wide analysis of serum samples was performed by small-RNA Sequencing on a NextSeq500 instrument (Illumina), sequencing in single-end mode 1x75bp and 8 bp for the i7 indices. Sample demultiplexing was conducted with BaseSpace. Primary bioinformatic analysis was performed with GeneGlobe® Data Analysis Center. The reads were processed with QIAseq® miRNA Primary Quantification tool (legacy pipeline). This analysis includes alignment and miRNAs, piRNAs, tRNAs and other RNAs quantifications. The complete description of all the steps can be found at the following link: https://ngsdataanalysis2.qiagen.com/handbooks/HB-2608-001_SP_Qseq_miRNA_Quantification_1118_WW_20181106_BA_12072018.pdf. UMI counts were normalized by considering a size factor for each sample.

To estimate the size factors, we considered the median of the ratios of observed counts to those of a pseudo-reference sample, whose counts can be obtained by considering the geometric mean of each gene across all samples [15]. Then, to transform the observed counts to a common scale, the observed counts in each sample were divided by the corresponding size factor. To infer differential gene expression a Negative Binomial Model was build. Linkage between the variance and the mean was established by a locally regressed non-parametric smooth function of the mean. The Benjamini-Hochberg (BH) procedure for multiple testing was used to obtain adjusted *P*-values. Average expression of standardized counts of the gene signature was used to fit a binomial model. Prediction scores from the classifier were then considered to evaluate the true positive rate (sensitivity) and the false positive rate (1-specificity) in a ROC curve. Performance of the curves was assessed by calculating the Area Under Curve (AUC) with 1000 bootstrap replicas for computation of the confidence bounds. Selection of miRNAs was based on different criteria. MiRNAs with AUC from a logistic regression higher than 0.70 in prediction of IDH mutation or miRNAs significantly involved in prognosis were selected for further analyses.

### Cell culture and RNA extraction

SW1783, U87MG and T98G were cultured in DMEM medium GlutaMAX™ Supplement (#10566016 Gibco) supplemented with 10% heat-inactivated foetal bovine serum (FBS; #10270106 Gibco), and 1% penicillin–streptomycin (P/S; #15070063 Gibco). BT142 was grown in NeuroCult NS-A proliferation medium (#05751, Stem Cell Technologies) supplemented with 20 ng/mL recombinant human Epidermal Growth Factor (EGF; #AF-100-15 PeproTech), 100 ng/mL recombinant human Platelet-Derived Growth Factor-AA (PDGF-AA; #500-P46 PeproTech), 20 ng/mL recombinant human Fibroblast Growth Factor (FGF; #100-18B PeproTech) and 2 μg/mL Heparan sulfate (#H4777, Sigma). All cell lines were cultured in a humidified atmosphere at 37 °C, 5% CO_2_. The identity of cell lines was confirmed by short tandem repeat profiling. RNA extraction from cell conditioned medium (extracellular fraction), starting from 400 μL, was performed using the miRNeasy Serum/Plasma Advanced kit (#217204, Qiagen). For culture medium collection, cells were seeded and after 24 h the medium was replaced with Serum-Free Medium (SFM). Culture medium was centrifuged at 2000 g for 10 min, filtered using 0.22 μm polyether-sulfonate low-protein binding filters and aliquoted. Total RNA was isolated from cells (intracellular fraction) by TRIsure reagent (#BIO-38033 Bioline, Meridian Bioscience) and quantified by NanoDrop ND-1000 spectrophotometer (ThermoFisher Scientific).

### Functional assays

Cell viability was evaluated with Cyto3DTM Live-Dead Assay Kit (#BM01, TheWell Bioscience) following manufacturer's instructions. Several images were taken by using Bio-Rad ZOE fluorescent cell image under a phase-contrast microscope (Biorad).

Proliferation was assessed using the 3-(4,5-dimethylthiazol-2-yl)-2,5-diphenyltrazolium bromide (MTT) assay (#M2003, Sigma). Briefly, MTT solution was added to transfected cells. After 3 h (37 °C) isopropanol solution was added. The absorbance was measured at 570 nm (microplate reader; Thermo Lab systems Multiskan EX).

For cell cycle analysis, cells were permeabilized in a solution of 0.1% Triton X-100, 0.1% sodium citrate, and stained with 50 μg/mL propidium iodide. DNA content was determined by flow cytometry using Cytoflex LX flow cytometer (Beckmann Coulter Life Sciences).

Apoptosis was evaluated by Annexin V staining (Apoptosis Detection Kit FITC; Invitrogen).

For cell migration, cells (3 × 10^4^) were seeded on 8.0 μm pore sized membranes (#662638; Greiner Bio-one) and allowed to migrate for 48 h. Serum-free medium was added to the lower chamber. For 2D invasion, cells (5 × 10^4^) were seeded on 8.0 μm pore sized membranes (#662638; Greiner) coated with Cultrex Reduced Growth Factor Basement Membrane Matrix (#3433–005-01, R&D Systems) and left to invade for 72 h. Serum-free medium was added to the lower chamber. For both migration and invasion assays, cells on the upper part of the membrane were scraped using a cotton swab, and the migrated cells were stained using Three-Step Stain Set (#3413653, ThermoScientific).

For 3D invasion assay, cell spheroids were generated by plating 100 cells for 48 h in 3D Culture Qualified 96 Well Plate (#3500–096-K, Greiner). Then, spheroids were embedded into Cultrex Reduced Growth Factor Basement Membrane Matrix (#3433–005-01, R&D Systems) according to the manufacturer’s instructions. After 1 h at 37 °C, serum-free media was added to spheroids and then allowed to invade for 72 h. Transmigrated and spheroids cells were photographed by using Bio-Rad ZOE fluorescent cell imager (Biorad).

Invadopodia activity was evaluated by gelatin degradation assay as described previously [16], using QCM Gelatin Invadopodia Assay (Green) (#ECM670, Millipore). Experiments were done in triplicates by imaging > 10 fields of view and > 5 cells in each sample. Scale bar = 10 μm.

### Cell lines transfection

For transient miRNAs overexpression, cell lines were transfected with miR-1-3p, miR-26a-1-3p, miR-487b-3p mimics (Dharmacon) or negative control mimic (MirVana miRNA Negative control #1, Invitrogen) using the Lipofectamine RNAiMAX (#13778075, Invitrogen) according to manufacturer’s instructions. For transfections with the combined miRNA-mimics (miR-1-3p, miR-26a-1-3p, miR-487b-3p), a final concentration of 0.5 nM was used for each one, reaching a total 1.5 nM concentration of the three-miRNA mimic combination. Conversely, to assess the functional effects of the single miRNAs, 0.5 nM of the miRNA-mimic of interest was used together with 1 nM of negative control in order to reach the same total oligonucleotide concentration.

For transient gene expression, glioma cells were transfected with *SH3PXD2A*, *SH3PXD2B* or *EFHD2* expression vectors (pRP-EGFP/Puro-CAG > FLAG-hSH3PXD2A, −hSH3PXD2B and -hEFHD2; VectorBuilder) by Lipofectamine 3000 reagent (#L3000001, Invitrogen), according to manufacturer’s instructions. After 24 h cells were transfected with miRNA mimics as above described and plated for migration and invasion assays.

For Luc assay, the target sites for miR-1-3p, miR-26a-1-3p or miR-487b-3p in human *SH3PXD2B* 3’UTR (NM_001017995.3), *SH3PXD2A* (NM_001394015.1) and *EFHD2* (NM_024329.6), and their mutants were cloned into pGL3 Control vector (#E1741, Promega) or into psiCHECK2 vector (#C8021, Promega) downstream of the luciferase gene. Human HEK-293 T (RRID: CVCL_4U22) cells were transiently co-transfected by Lipofectamine 2000 (#11668019, Invitrogen), with 800 ng of firefly luciferase reporter plasmid containing wild-type or mutant 3’UTRs of *SH3PXD2B*, *SH3PXD2A* or *EFHD2* and of either the miRNA mimics hsa-miR-1, hsa-miR-26a-1 and hsa-miR-487b (20 pmol, Dharmacon) or control (Ctrl)-mimics RNA oligonucleotides (20 pmol, ThermoScientificBio). 48 h post-transfection cells were lysed and luciferase activity quantified using the Dual Luciferase Reporter kit (#E1910, Promega), according to the manufacturer’s instructions.

### Western blot

Extracts from transfected cells were subjected to immunoblot analysis as described [17]. Proteins of interest were detected using: anti-PARP (#9542 Cell Signaling), anti-TKS4 (#A303-436A, Bethyl Laboratories), anti-TKS5 (#09–268 Merck Millipore), anti EFHD2 (#PA5–61846 Invitrogen) and anti-PCNA (#SCFL261, Santa Cruz) antibodies. Anti-Beta-Actin (#3700, Cell Signaling) was used as housekeeping protein to normalize.

### Reverse transcription and quantitative real-time-PCR (qRT-PCR)

Analysis of mature miRNAs was performed using TaqMan® MicroRNA Reverse Transcription Kit followed by qRT–PCR according to manufacturer’s instructions (#4366597 Applied Biosystems) on an ABI 7900 Real Time PCR System and SDS 2.2.2 software. Samples were normalized to RNU44 small RNA.

RNA quantification of *SH3PXD2B, SH3PXD2A* and *EFHD2* was performed using SYBR Green-based qRT-PCR as described [18] using specific primers, available upon request. GAPDH gene expression was used as endogenous control.

### Bioinformatic and statistical analysis

Standardized TCGA and CGGA data were obtained from the Broad Institute TCGA Genome Data Analysis Center (2016, 10.7908/C11G0KM9) and The Chinese Glioma Genome Atlas database (http://www.cgga.org.cn/), respectively. Differential expression of miRNAs between subgroups of samples were evaluated by the two-sided Wilcoxon rank sum test. Survival analyses were performed by using the Kaplan-Meier method. Differences between curves were assessed by the log-rank test. Patients with high and low signal intensity for a specific miRNA signature were defined by considering positive and negative z-score values of the mean intensity values of the miRNAs. A Cox proportional hazard regression model was fitted to include clinical variables. Target prediction of selected miRNAs and gene set enrichment analysis were defined by using the last version of miRWalk web tool (http://mirwalk.umm.uni-heidelberg.de/). All the analyses were conducted with MATLAB R2020b. The significance of intergroup differences was estimated with the Student’s t-test, or Mann-Whitney-U Test as appropriate. All results are shown as the mean ± standard deviation of the mean (SD) of 3 independent experiments. Statistical significance was considered to be *p* ≤ 0.05.

## Results

### Global serum miRNA profiling in glioma patients with different IDH mutation status

**Fig. 1:**
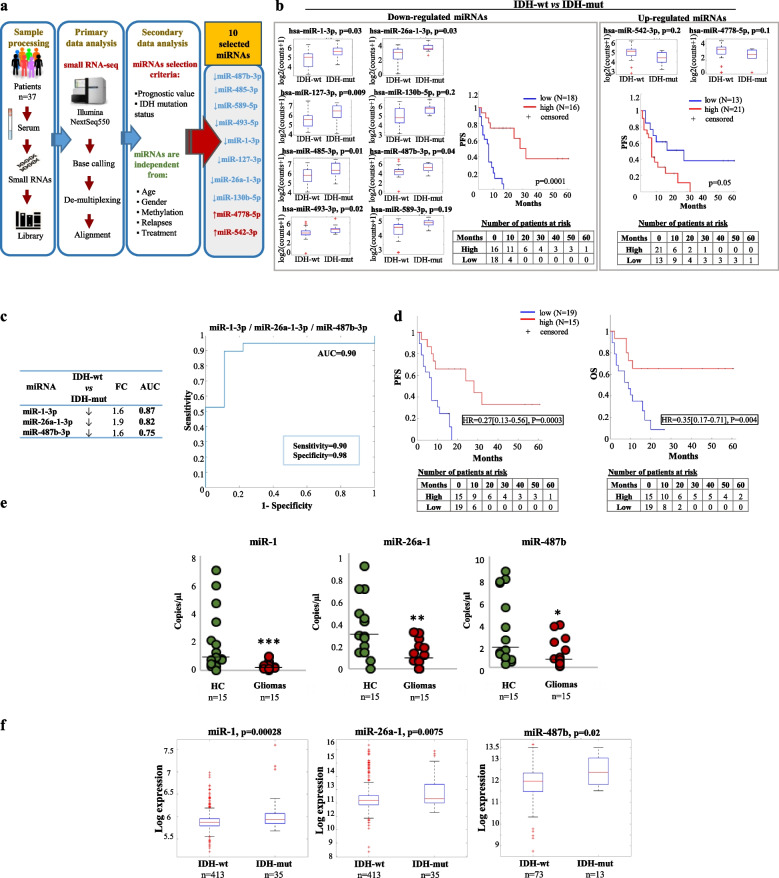
Genome-wide analysis and selection of a circulating miRNA-signature deregulated in glioma patients based on IDH-mutation. **a** Schematic representation of the experimental workflow for the global miRNA profiling by small RNA-seq; **b**) Box-plots and Kaplan-Meier curves based on the indicated miRNA expression levels in serum samples of glioma patients. In the box-plots serum miRNA levels in IDH-wild type (wt) vs. IDH-mutant (mut) patients are reported as Log2, boxes define the 25th and 75th percentiles, the horizontal line in the boxes indicates the median, and bars define the minimum and maximum values. The Kaplan-Meier plots indicate the relation between the miRNAs downregulated (l*eft panel*) or upregulated (*right panel*) in IDH-wt vs. IDH-mut patients and the PFS. Patients with high and low signal intensity for a specific miRNA signature were defined by considering positive and negative z-score values of the mean intensity values of the miRNAs. **c** Fold change expression (FC) and area under the ROC curve (AUC) of the indicated miRNAs and ROC curve plotted for diagnostic potential and discriminatory accuracy of serum miR-1/−26a-1/−487b combination. **d** Kaplan-Meier survival plots for PFS and OS based on miR-1/−26a-1/−487b expression levels. **e** Scatter plots of the expression levels of the indicated miRNAs, analysed by digital PCR, in serum samples of glioma patients vs. healthy controls (HC). **f** Box-plots of the indicated miRNA expression levels in IDH-wt vs. IDH-mut tumour tissue samples from a LGG dataset of the TCGA (*left and middle panels*) and from a GBM dataset of the CGGA (*right panel*); values are reported as Log2. Boxes define the 25th and 75th percentiles. The horizontal line in the boxes indicates the median and the edges of the box are the 25th and 75th percentiles * = *p* ≤ 0.05, ** = *p* ≤ 0.01, *** = *p* ≤ 0.001

To assess whether circulating miRNAs can mirror the mutation status of IDH genes, the serum-miRNome of glioma patients was analysed by small RNA-Sequencing in preoperative serum samples of a cohort of 37 glioma patients with different IDH mutation status (*n* = 27 IDH-wild type and *n* = 10 IDH-mutant), matched by gender and age (Table 1; Fig. 1a). Ten serum miRNAs (miR-1-3p, miR-26a-1-3p, miR-127-3p, miR-130b-5p, miR-485-3p, miR-487b-3p, miR-493-5p, miR-542-3p, miR-589-5p and miR-4778-5p) were then selected based on the Area Under Curve (AUC) from a logistic regression higher than 0.70 in prediction of IDH mutation, and their prognostic value (logrank test < 0.05). These selection criteria resulted in 8 miRNAs down-regulated (≥1.3 folds; Fig. 1b *left panel*) and 2 miRNAs up-regulated (≥1.4 folds; Fig. 1b *right panel*) in IDH-wild type (IDH-wt) compared to IDH-mutant (IDH-mut) samples. Kaplan-Meier curves and log-rank test showed that the expression of these miRNAs significantly correlated with the Progression Free Survival (PFS), taking into account both the miRNAs down-regulated in IDH-wt (hazard ratio: 0.24, 95% CI: 0.12–0.47 Fig. 1b *left panel*) and those up-regulated (hazard ratio: 2.1, 95% CI: 1.16–3.83; Fig. 1b *right panel*), as expected considering their correlation with the IDH mutation status.

To evaluate the diagnostic potential and discriminatory accuracy of the selected serum-miRNAs in glioma patients, ROC curves were established. Our analysis revealed that the expression levels of each single miRNA of the signature, were robust in discriminating patients with IDH-wt versus IDH-mut, with an AUC value ranging from 0.75 to 0.87 (Fig. S1). Next, different combinations of the selected miRNAs were considered to evaluate whether they could provide an improvement of the diagnostic accuracy. As shown in Fig. 1c, the combination of miR-1-3p, miR-26a-1-3p and miR-487b-3p, each one upregulated in IDH-mut subjects, led to a substantial improvement of the diagnostic performance (AUC 0.90; 95% CI = 0.7381–0.9689), compared to each single miRNA of the signature. Moreover, this restricted three serum miRNA signature is significantly correlated with both Overall Survival (OS) and PFS of glioma patients (Fig. 1d). To further confirm the diagnostic potential of the identified signature, we evaluated its expression in serum samples of 15 glioma patients (8 IDH-wt, 7 IDH-mut) and 15 age and gender-matched healthy subjects (Table 1).

As shown in Fig. 1e, all signature members are significantly downregulated in the serum of glioma patients compared to controls, thus supporting their diagnostic value.

To support the data obtained in patient serum, we have analyzed the expression of miR-1-3p, miR-26a-1-3p and miR-487b-3p in glioma tissues datasets. Since in The Cancer Genome Atlas (TCGA) database miRNA data were only available for low grade gliomas (LGG; 448 LGG that include 413 IDH-wt patients and 35 IDH-mut), we examined also the Chinese Glioma Genome Atlas (CGGA) where miRNA data from 86 high grade glioblastoma (GBM) patients are reported (73 IDH-wt patients and 13 IDH-mut). In the TCGA dataset, we found that in LGG the regulation of miR-1-3p and miR-26a-1-3p, but not miR-487b-3p, were consistent with our data in serum, being those miRNAs down-regulated in IDH-wt patients compared to IDH mutant subjects. Instead, the analysis of the CGGA dataset showed that in GBM, the miR-487b was significantly upregulated in IDH-mut patients compared to IDH-wt, in line with the results of our patient cohort (Fig. 1f).

To evaluate whether glioma cell lines with different IDH mutation statuses may recapitulate the results obtained in glioma patients, the expression levels of miR-1/miR-26a-1/miR-487b were assessed in the cell fraction and in the paired extracellular conditioned medium of SW1783 (astrocytoma III, IDH-wt) and BT142 (oligoastrocytoma III, the only IDH-mut glioma cell line commercially available in the ATCC collection [19]) glioma cell lines. We chose these cell lines because, although they have different IDH mutation statuses, both of them derived from grade III gliomas. As shown in Fig. S2, the baseline expression level of two out of three miRNAs of the signature (miR-26a-1-3p and miR-487b-3p) is downregulated in the IDH-wt compared to the IDH-mut cells (intracellular fraction), while all the miRNAs of the signature are released at lower levels in the conditioned medium (extracellular fraction) of the IDH-wt glioma cell line compared to the IDH-mut cells. Interestingly, we also found that the value of extracellular to intracellular expression ratio for two out of three miRNAs (miR-1-3p and miR-26a-1-3p) is significantly higher in IDH-mut BT142 cells (Fig. S2). These data suggest that IDH-mut cells have a higher rate of these miRNA release in the medium of IDH-mut tumours compared to the IDH-wt counterpart, as observed in glioma patient serum.

### Impact of a three-miRNA signature (miR-1, miR-26a-1 and miR-487b) on glioma biology

**Fig. 2:**
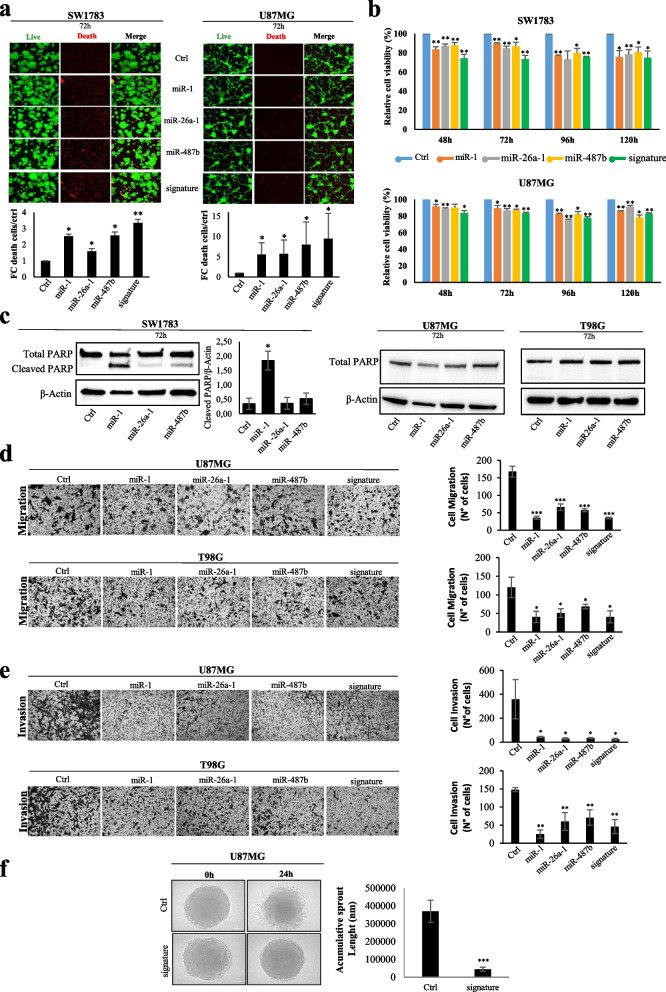
Biological effects of miR-1-3p/−26a-1-3p/−487b-3p ectopic expression in IDH-wt glioma cells. Analysis of **a**) Cell viability by Cyto3D™Live-Dead assays and **b**) cell proliferation in SW1783 and U87MG cell lines after 48, 72, 96 and 120 hours of transfection with individually or combined indicated miRNA-mimics. Cell viability values are expressed as fold change of death cells respect to control and proliferation as optical density at 750 nm wavelength, proportional to the quantity of metabolically active cells. c) Western blots of a representative experiment of total and cleaved-PARP in the indicate glioma cells after 72 h of miR-1, miR-26a-1 and miR-487b ectopic expression. Actin was used as loading control within the same sample and expressed as fold changes compared to control. Densitometric analysis by imageJ software for SW1783 glioma cells is shown. Analysis of 2D **d**) migration and **e**) invasion, by Transwell systems, of high-grade glioma cells 72 h after single or combined transfection with the indicated miRNAs. **f**) 3D-invasion assay with spheroids of glioma high-grade cells transfected with miRNA-mimic combination or negative control (Ctrl). The quantification of sprouts length 24 h after spheroid formation is reported. All the values are reported as mean of at least three experiments. Error bars indicate the standard deviation. * = *p* ≤ 0.05, ** = *p* ≤ 0.01, *** = *p* ≤ 0.001

To investigate the role of the miRNA signature in gliomas, we assessed the consequences of its overexpression in glioma IDH-wt cells. As shown in Fig. 2, the individual overexpression of the three miRNAs negatively affects cell viability (Fig. 2a) and proliferation (Fig. 2b) mainly in the grade III IDH-wt SW1783 cells, while in the higher grade U87MG, the effect is more pronounced when the entire signature is overexpressed in combination. Indeed, in both the cell lines the combined overexpression of the three-miRNAs led to a stronger inhibition of both proliferation and viability (Fig. 2a, b). To further support these data, we evaluated the expression levels of the Proliferating Cell Nuclear Antigen (PCNA) as a proliferation marker [20]. The results show that the PCNA expression is significantly inhibited upon the overexpression of the signature members both in SW1783 and in U87MG cell lines (Fig. S3). The DNA content analysis revealed that in low-grade SW1783 glioma cells but not in high-grade glioma cells (U87MG), miR-1 overexpression causes a decrement of G2 phase and an increase of sub-G1/G0 pick. These data suggest that miR-1 could cause a block of the cell cycle progression to G2 and a concomitant increase of apoptosis (Fig. S4) whereas the other miRNAs of the signature do not show significant changes in the cell cycle distribution probably due to their milder effect on proliferation (Fig. 2b).

Next, we evaluated the effect on apoptosis analysing the cleavage of PARP protein. We found that levels of cleaved-PARP are increased mainly by miR-1 overexpression and to a lesser extent by miR-487b (5.3-fold and 1.5-fold increase, respectively; Fig. 2c). Of note, we observed these pro-apoptotic effects only in grade III astrocytoma IDH-wt SW1783 cell line, while no effects are observed in grade IV U87MG cells. This was further confirmed in T98G cells, another GBM cell line, suggesting differences in the molecular mechanisms controlling apoptosis depending on the cell context and tumour grade (Fig. 2c). In support of these data, as shown in the Fig. S5, an increase of Annexin V positive cells is only observed in SW1783 after miR-1 overexpression. Finally, experiments aimed at assessing miRNAs impact on glioma migration and invasion were performed on high-grade U87MG and T98G cells, since the lower grade SW1783 cell line had a low basal invasion capability (data not shown). Results show that the individual overexpression of all signature members, as well as their combination, led to a significant suppression of both migration (Fig. 2d) and invasion (Fig. 2e, f).

### Identification and validation of the miRNA-signature targets

**Fig. 3:**
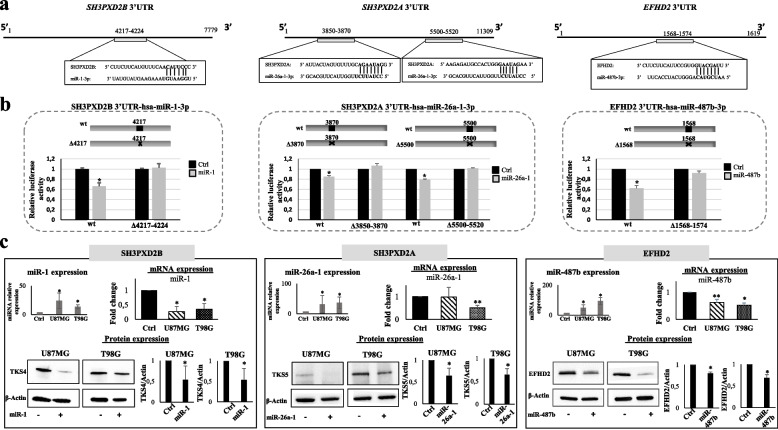
Identification and validation of *SH3PXD2B, SH3PXD2A* and *EFHD2* as miR-1-3p, miR-26a-1-3p and miR-487b-3p direct targets. **a** Schematic representation of putative binding sites for miR-1, miR-26a-1 and miR-487b in the 3’UTR of the *SH3PXD2B*, *SH3PXD2A and EFHD2* genes, respectively; **b)** Firefly luciferase activity in recipient cells after transient co-transfection with *Renilla* luciferase reporter plasmid containing the wild-type (wt) or the indicated mutant (mut) sequences of miR-1-3p, miR-26a-1-3p and miR-487b-3p target sites, and the relative miRNA-mimic or ctrl-mimic. Results are expressed as fold activation relative to the basal activity of pGL3 empty control (ctrl); **c)** *SH3PXD2B-*, *SH3PXD2A-* and *EFHD2*-mRNA and related protein expression levels in the indicated IDH-wt glioma cells after the indicate miRNAs overexpression. All the values are reported as mean of at least three experiments. Error bars indicate the standard deviation. * = *p* ≤ 0.05, ** = *p* ≤ 0.01

To gain more information on the functional role of miR-1, miR-26a-1 and miR-487b, we performed an in silico analysis of its putative target genes using the miRWalk3.0 database. Since the most marked functional effect observed upon miRNA signature ectopic expression in glioma cells was the inhibition of migration and invasion, from the resulting target genes, we selected those which are reported to be involved in cell motility, migration and invasion. In particular, we focused our attention on *SH3PXD2B*, *SH3PXD2A* and *EFHD2* as putative targets of miR-1, miR-26a-1 and miR-487b, respectively. *SH3PXD2A* and *B* encode for the Tyrosine Kinase Substrate with five (TKS5) and with four (TKS4) SH3 Domains, respectively. TKS5 and TKS4 are scaffold proteins that belong to the same superfamily and are involved in the regulation of cell adhesion and migration [13, 21, 22]. EFHD2 is a Ca^2+^ binding adapter protein that regulates the actin bundling and is involved in cell migration, too [14, 23–25]. *SH3PXD2B-*mRNA and *EFHD2*-mRNA 3’UTR regions contain one putative binding site for the related targeting miR-1 and miR-487b, respectively, while the *SH3PXD2A*-mRNA 3’UTR contains 2 putative miR-26a-1-3p target sites (3870 and 5500; Fig. 3a). To assess whether the selected genes are direct target of the miRNA signature, a human 3’UTR fragment with or without their target seed sequences (Fig. 3b) was cloned downstream of the firefly luciferase reporter gene and co-transfected with the related miRNA mimics in 293 T recipient cells. The relative luciferase activity of the reporter with wild-type 3’UTR was decreased by 35% for miR-1 (*SH3PXD2B* 3’UTR), 15 and 20% for miR-26a-1, considering the two putative binding sites (*SH3PXD2A* 3’UTR), and 38% for miR-487b (*EFHD2* 3’UTR). As expected, there was no decrease in luciferase activity of all the mutant reporters suggesting that the selected target genes are novel and direct targets of the miRNA signature. To further confirm these results, we evaluated the expression levels of *SH3PXD2B*, *SH3PXD2A* and *EFHD2* mRNAs and encoded proteins in IDH-wt glioma cells (U87MG and T98G) overexpressing miR-1, miR-26a-1 and miR-487b, respectively. As shown in Fig. 3c, the protein levels of the selected targets are decreased upon miRNAs overexpression in both cell lines. In agreement with these data, we found a decrease of the respective mRNAs levels, except for the expression of *SH3PXD2A* mRNA that decreased after miR-26a-1 mimic transfection in T98G but not in U87MG cells. However, since the *SH3PXD2A* encoded protein (TKS5) decreases after miR-26a-1 overexpression in both the cell lines, we hypothesize that in T98G cells miR-26a-1 impairs TKS5 expression influencing both mRNA stability and translation while impacting only on mRNA translation in U87MG. Altogether, these findings strongly suggest that the signature could influence migration and invasion through the combined modulation of TKS4/5 and EFHD2 scaffold proteins.

**Fig. 4:**
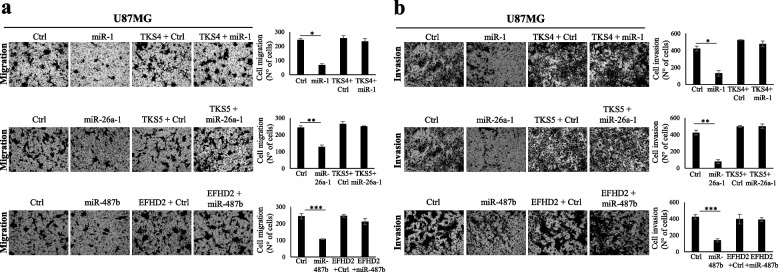
*SH3PXD2B, SH3PXD2A* and *EFHD2* target genes mediate the signature functional impact on cell migration and invasion **a**) Migration and **b**) Invasion, by Transwell systems, of high-grade glioma cells 72 h after transfection with the indicated miRNAs and genes. All the values are reported as mean of at least three experiments. Error bars indicate the standard deviation. * = *p* ≤ 0.05, ** = *p* ≤ 0.01, *** = *p* ≤ 0.001

The involvement of the identified target genes in mediating the signature functional impact on cell migration and invasion, was also evaluated by rescue experiments. As shown in Fig. 4, co-transfection of U87MG glioma cells with the signature targets and the related targeting miRNA mimics significantly counteract the decrease of cell migration (Fig. 4a) and invasion (Fig. 4b) observed upon miRNAs ectopic expression. The same results were also obtained in the T98G cell line (data not shown).

### Impact of the miRNA-signature on invadopodia formation

**Fig. 5:**
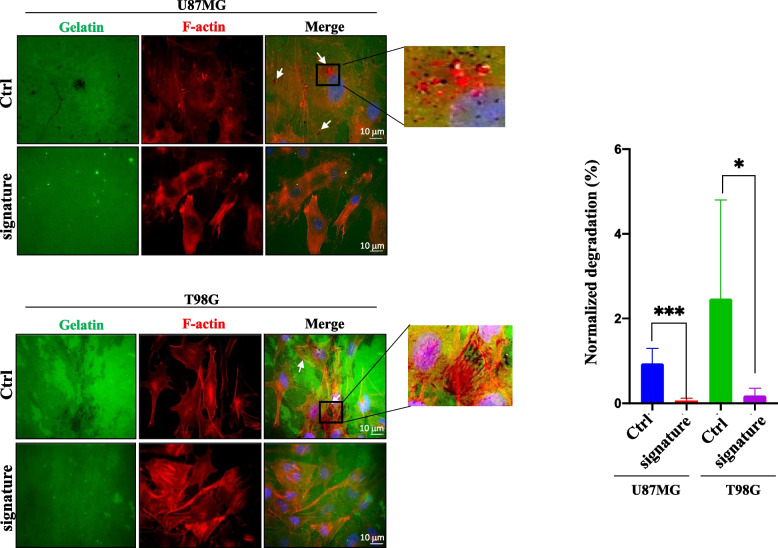
Inhibition of invadopodia activity by miR-1-3p, miR-26a-1-3p and miR-487b-3p signature. Immunofluorescence analysis of U87MG and T98G cells, transfected with the three-miRNA signature or negative control, plated onto gelatin matrix (green) and cultured for 72 h. Representative images show F-actin structures (red), and nuclei (blue, DAPI). Co-localization of areas of degraded gelatin (black) and F-actin structures is shown in merged images (pink dots indicated by arrows) and reported as zoomed pictures. Experiments were done in triplicates by imaging > 10 fields of view and > 5 cells in each sample. Scale bar = 10 μm Histograms indicate the means±SD of normalized degradation area percentage of cells. * = *p* ≤ 0.05, *** = *p* ≤ 0.001

TKS4 and 5 are large SRC Homology 3 (SH3) domain containing proteins that act as docking sites for several signalling molecules regulating podosomes and invadopodia formation and activity [13, 26]. Also EFHD2 is described to be involved in cell motility and its ectopic expression promotes the formation of membrane structures such as lamellipodia and invadopodia [14, 25]. Thus, we investigated whether inhibition of the expression of these protein by the miRNA signature could have an effect on the formation of these protruding membrane structures. To this end, we analysed changes in the invadopodia activity in U87MG and T98G cells before and after the combined overexpression of the signature. Of note, our data show a complete inhibition of invadopodia in miRNA-overexpressing cells (Fig. 5), thus suggesting that the identified miRNA-signature impairs cell invasion by a suppression of invadopodia activity.

## Discussion

The identification of non-invasive markers represents an important goal in improving the management of brain tumour patients [6]. Thus, the search for blood circulating biomarkers has become a field of study drawing the interest of the scientific community [2]. Among the circulating molecules, miRNAs are the most investigated biomarkers mainly due to their frequent alteration in cancer and their high-stability in biofluids [2, 27–29]. Despite the large number of miRNAs examined as single biomarkers in gliomas [29], only a few comprehensive analyses of the entire circulating-miRNA repertoire in glioma patients have been performed [30, 31].

In this study, using an unbiased high-throughput next-generation sequencing and an integrative bioinformatics pipeline, we have identified 10 -serum miRNAs able to stratify glioma patients on the basis of the mutation status of IDH genes with high specificity and sensitivity. Many of the identified miRNAs play a role in glioma biology suggesting their importance as putative biomarkers for this pathology [30, 32–34]. Among the identified miRNAs, we focused our attention on the combination of miR-1-3p/miR-26a-1-3p/miR-487b-3p since it led to an improvement in the diagnostic performance, compared to each single miRNA, thus supporting the value of a combined approach for the application of circulating miRNAs as diagnostic biomarkers. Moreover, we have also demonstrated that the selected miRNAs act as tumour suppressors in IDH-wt gliomas by exerting adverse effects on cell viability and proliferation in vitro. Of note, the combined modulation of all signature members led to a more prominent impact on glioma biological functions, especially in high-grade GBM cells, suggesting the value of the identified signature in toto as a putative therapeutic target as well as supporting the rationale and feasibility of combinatorial miRNA strategies for anticancer therapies [9]. Previous studies demonstrated the involvement of miR-1-3p in brain tumour biology. In particular, it has been described as a tumour suppressor in many types of cancer, including gliomas, as it inhibits the epithelial to mesenchymal transition by directly targeting fibronectin [35–38]. On the other hand, only little information is available on the possible role of miR-26a-1-3p and miR-487b-3p in the pathogenesis of these tumours [39–42].

Here we show, as the most relevant biologic effect, that all the signature members significantly inhibit the migration and invasion in GBM cells. In this regard, we have identified and validated *SH3PXD2B*, *SH3PXD2A* and *EFHD2* as direct targets of miR-1-3p, miR-26a-1-3p, and miR-487b-3p, respectively. The first two genes encode for TKS4 and TKS5 adaptor proteins, respectively, that belong to the p47^*phox*^ superfamily and play a role as orchestrators of key signalling events regulating the assembly and activity of membrane-located structures known as invadopodia [13]. These are dynamic actin-dependent protrusions produced by many types of cancer cells by which they can adhere to and subsequently degrade extracellular matrix via the action of various transmembrane and secreted proteases [13, 26, 43]. TKS4 and TKS5 have been observed to play a role in the development and progression of several cancers [21, 44, 45]. In glioma tissues both TKS4 and 5 are described as overexpressed compared to normal brain [46] and TKS5 expression has also been associated with poor glioma patient survival [47].

*EFHD2* gene, target of miR-487b, is a Ca^2+^ binding protein that shows a predominant level of expression in the central nervous system [48], its deregulation is linked to the development of neurodegenerative diseases and synaptic disorders [48, 49]. In cancer EFHD2 has been associated with tumour invasion thus showing a potential interest as therapeutic target [14, 23–25] but the molecular mechanisms at the basis of these observations are less known than those related to TKS4 and TKS5. Indeed, both these scaffold proteins are involved in EGF signalling by regulating the actin cytoskeleton via EGFR and Src activity [13, 50]. EGFR/Src signalling is often aberrantly upregulated in glioma, promoting cell proliferation and migration where it is associated with tumour progression and neoangiogenesis [51]. For these reasons, many clinical trials are focused on the use of Src inhibitors for glioma treatment [52]. Lastly, it has been demonstrated that TKS4 and 5 may have a partially overlapping function in invadopodia regulation [53] thus their simultaneous inhibition induced by the identified miRNA-signature could counteract possible rescue mechanisms put in place by tumour cells and synergistically strengthen the inhibition of glioma invasive phenotype elicited by the signature.

EFHD2 also controls the actin cytoskeleton dynamics and regulate actin bundling in different tissues across several species [23, 24]. EFHD2 modulating activity on cell invasion has been described in some tumours such as melanoma, where it mediates the formation of motile protrusions in association with actin and the Rho family of GTPase [54], while the role of EFHD2 in brain tumours is less known. In this paper, for the first time, at least to our knowledge, we demonstrate that an impaired regulation of EFHD2 by miRNAs can sustain cell migration and invasion in a glioma context.

Altogether, our data suggest that *SH3PXD2A/B* and *EFHD2* may be of interest as putative therapeutic targets in potentially ‘druggable’ signalling pathways.

## Conclusions

This study highlights the potential for circulating miRNAs to be used for glioma subtyping and grading on the basis of clinically relevant molecular markers (IDH). The information obtained from miRNA profiling could complement that derived from tumour biopsy evaluations and facilitate treatment decision making and addressing patients to clinical trials with targeted therapies [55, 56]. In addition, the involvement of the identified miRNAs in several biological functions of glioma and their impact on common targets crucial for glioma migration and invasion suggest their value as putative therapeutic targets with potential implications for the development of miRNA-based complementary therapies.

## Supplementary Information

**Additional file 1.**

## Data Availability

The data underlying all findings of this study are publicly available at https://gbox.garr.it.

## Abbreviations

IDH: Isocitrate dehydrogenase
WHO: World Health Organization
miRNA: MicroRNA
cfDNA: Cell-free DNA
BBB: Blood brain barrier
IRE: Regina Elena National Cancer Institute
RT: Room temperature
ddPCR: Droplet-digital PCR
BH: Benjamini-Hochberg
AUC: Area Under Curve
FBS: Fetal bovine serum
P/S: Penicillin–streptomycin
SFM: Serum-Free Medium
PI: Propidium iodide
PBS: Phosphate Buffer Saline
SD: Standard deviation
qRT-PCR: Quantitative Real-Time-PCR
PFS: Progression Free Survival
OS: Overall Survival
TCGA: The Cancer Genome Atlas
LGG: Low grade glioma
CGGA: Chinese Glioma Genome Atlas
GBM: Glioblastoma
PCNA: Proliferating Cell Nuclear Antigen
TKS5: Tyrosine Kinase Substrate With Five SH3 Domains
TKS4: Tyrosine Kinase Substrate With Four SH3 Domains
SH3: SRC Homology 3
EFHD2: EF-Hand Domain Family Member D2
