# Supplementary material for: A diagnostic circulating miRNA signature as orchestrator of cell invasion via TKS4/TKS5/EFHD2 modulation in human gliomas
Source: J Exp Clin Cancer Res. 2023 Mar 17;42:66. doi: 10.1186/s13046-023-02639-8 (PMC10022260; doi:10.1186/s13046-023-02639-8)
Supplement: Supplementary file 1 — Additional file 1. [file 13046_2023_2639_MOESM1_ESM.pdf]

Figure S1

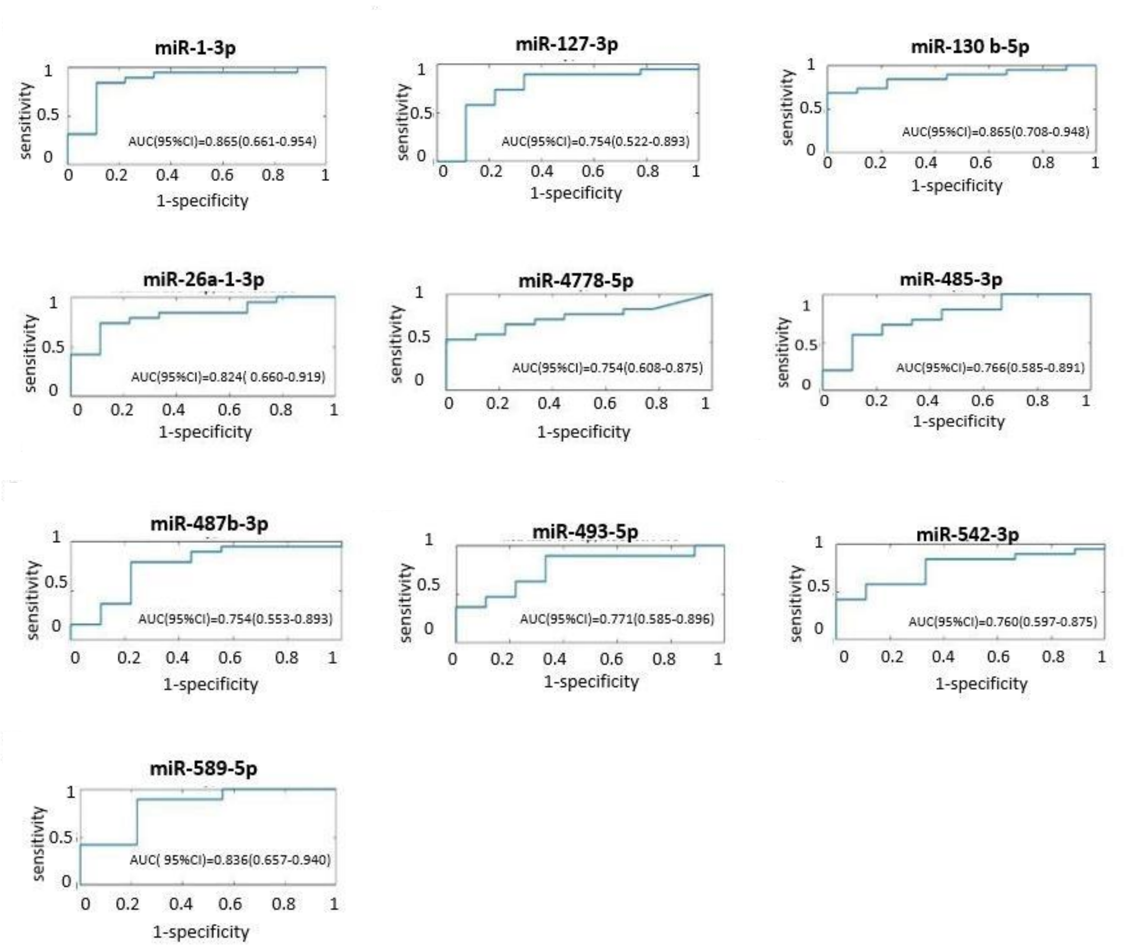

Figure S1. Accuracy of the indicated serum miRNAs in discriminating IDHwt form IDHmut patients between. ROC curves plots for the indicated miRNAs. AUC=Area under the curve; CI=confidence interval

Figure S2

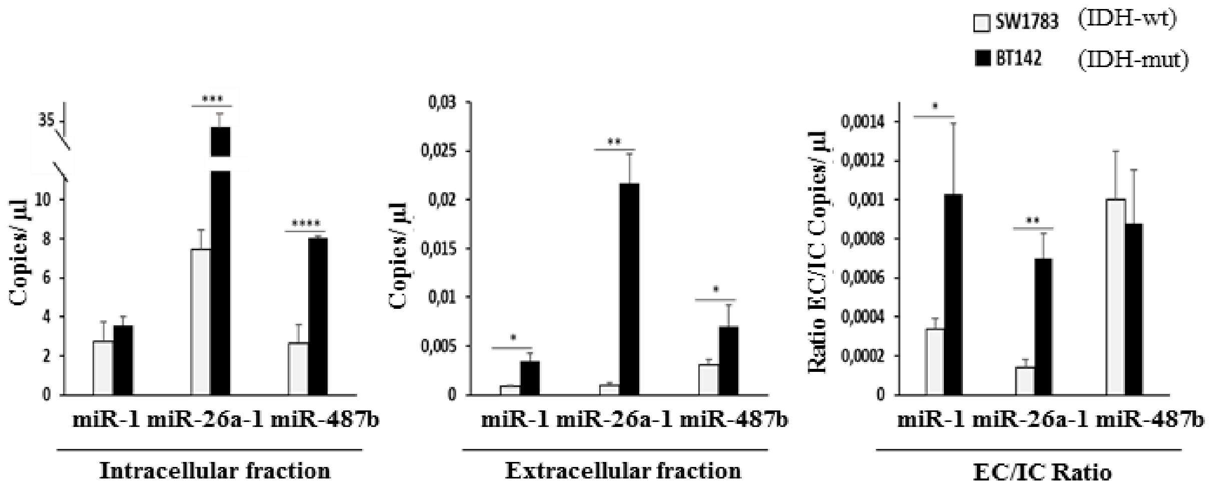

Figure S2. Expression levels of miR-1/-26a-1/-487b in glioma cell lines with different IDH mutation status. Expression levels of the indicated miRNAs in the intracellular (*left panel*: cells) and extracellular (*middle panel*: culture medium;) fractions of IDH-wt and IDH-mut glioma cell lines. Ratio of extracellular/intracellular (EC/IC) (*right panel*) expression levels of miR-1/-26a-1/-487b in IDH-wt (SW1783) and IDH-mut (BT142) glioma cells is indicated.

Figure S3

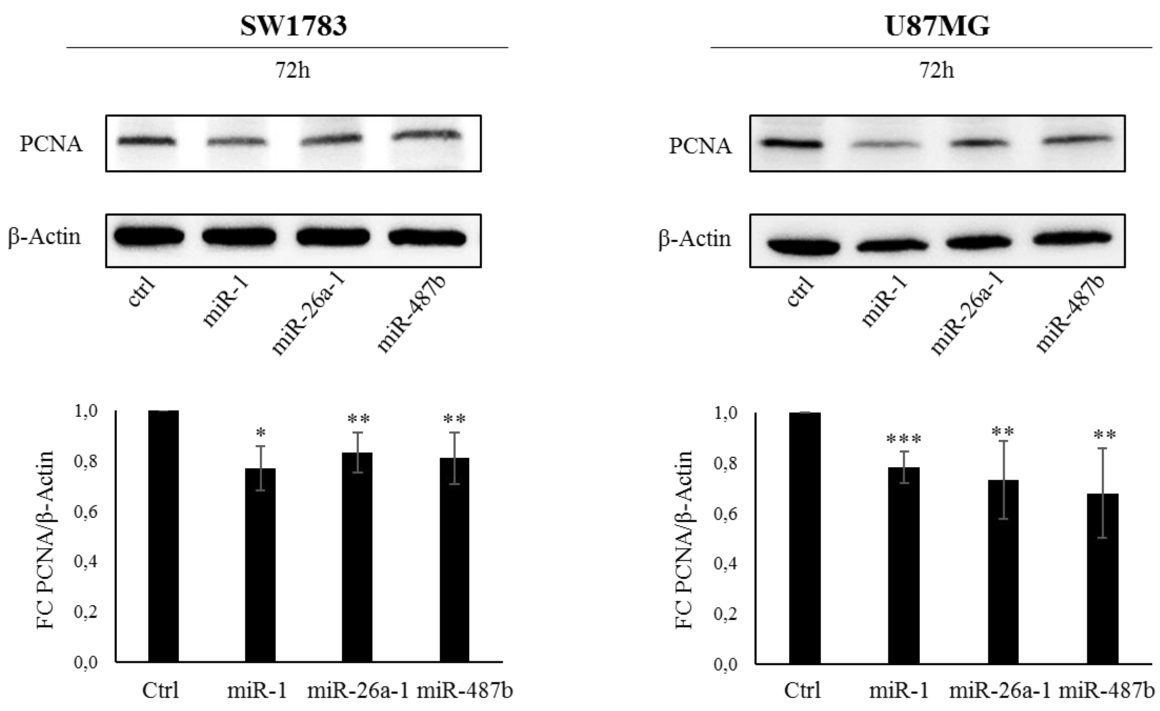

Figure S3. Analysis of the proliferation marker PCNA in miR-1, miR-26a-1 and miR-487b overexpressing cells. Western blots of a representative experiment of endogenous PCNA protein in the indicate glioma cells, 72h after transfection with miR-1, miR-26a and miR-487 or control RNA mimic. Actin was used as loading control within the same sample and expressed as fold changes compared to control. Densitometric analysis by imageJ software for SW1783 and U87MG glioma cells is shown (lower panel).

Figure S4

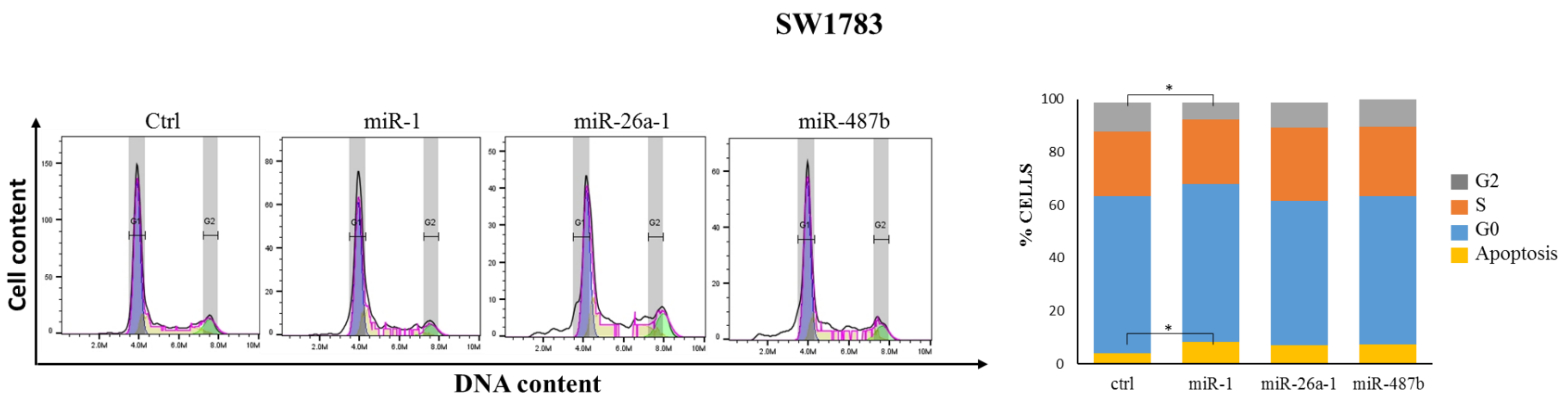

Figure S4. Cell cycle profile of SW1783 cell line after miR-1-3p/-26a-1-3p/-487b-3p ectopic expression. Analysis of DNA content using flow cytometry analysis of glioma cells 72h after miRNA-mimic transfection.

Figure S5

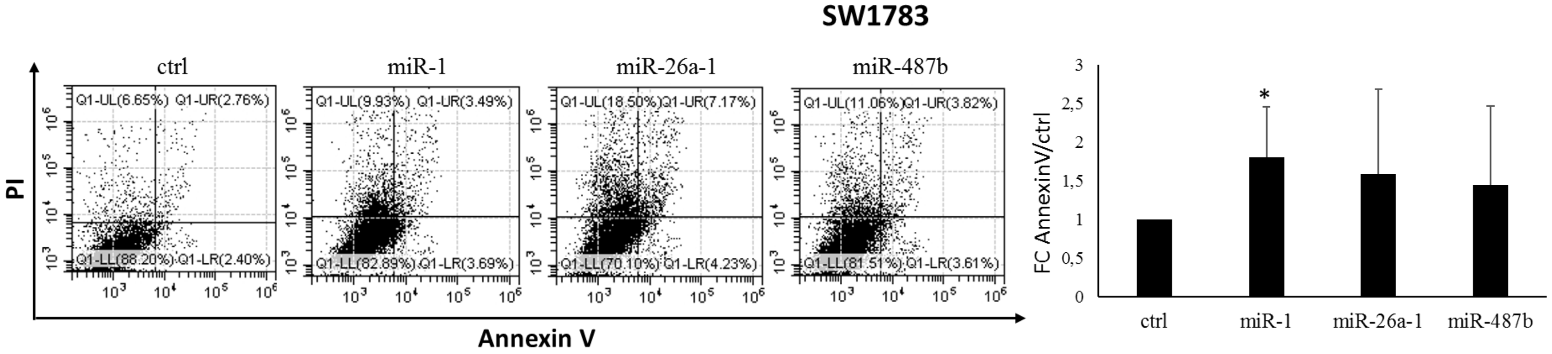

Figure S5. Analysis of Annexin V/PI in SW1783 cell line after miR-1-3p/-26a-1-3p/-487b-3p ectopic expression. SW1783 cells were analyzed 72h post-transfection with miRNA-mimics by flow cytometry after staining with Annexin V/PI. A representative experiment of the percentage of apoptotic cells (right lower quadrant, early apoptotic cells; right upper quadrant, late apoptotic cells) is shown. The graphic shows the fold change (FC) of apoptotic cells respect to the control cells.
